# Resting-State Brain and the *FTO* Obesity Risk Allele: Default Mode, Sensorimotor, and Salience Network Connectivity Underlying Different Somatosensory Integration and Reward Processing between Genotypes

**DOI:** 10.3389/fnhum.2016.00052

**Published:** 2016-02-17

**Authors:** Gaia Olivo, Lyle Wiemerslage, Emil K. Nilsson, Linda Solstrand Dahlberg, Anna L. Larsen, Marcela Olaya Búcaro, Veronica P. Gustafsson, Olga E. Titova, Marcus Bandstein, Elna-Marie Larsson, Christian Benedict, Samantha J. Brooks, Helgi B. Schiöth

**Affiliations:** ^1^Functional Pharmacology, Department of Neuroscience, Uppsala UniversityUppsala, Sweden; ^2^Section of Neuroradiology, Department of Radiology, Uppsala UniversityUppsala, Sweden; ^3^Department of Psychiatry, University of Cape TownCape Town, South Africa

**Keywords:** *FTO*, resting-state, MRI, obesity, SNP, default mode network, salience network, sensorimotor network

## Abstract

Single-nucleotide polymorphisms (SNPs) of the fat mass and obesity associated (*FTO)* gene are linked to obesity, but how these SNPs influence resting-state neural activation is unknown. Few brain-imaging studies have investigated the influence of obesity-related SNPs on neural activity, and no study has investigated resting-state connectivity patterns. We tested connectivity within three, main resting-state networks: default mode (DMN), sensorimotor (SMN), and salience network (SN) in 30 male participants, grouped based on genotype for the rs9939609 *FTO* SNP, as well as punishment and reward sensitivity measured by the Behavioral Inhibition (BIS) and Behavioral Activation System (BAS) questionnaires. Because obesity is associated with anomalies in both systems, we calculated a BIS/BAS ratio (BBr) accounting for features of both scores. A prominence of BIS over BAS (higher BBr) resulted in increased connectivity in frontal and paralimbic regions. These alterations were more evident in the obesity-associated AA genotype, where a high BBr was also associated with increased SN connectivity in dopaminergic circuitries, and in a subnetwork involved in somatosensory integration regarding food. Participants with AA genotype and high BBr, compared to corresponding participants in the TT genotype, also showed greater DMN connectivity in regions involved in the processing of food cues, and in the SMN for regions involved in visceral perception and reward-based learning. These findings suggest that neural connectivity patterns influence the sensitivity toward punishment and reward more closely in the AA carriers, predisposing them to developing obesity. Our work explains a complex interaction between genetics, neural patterns, and behavioral measures in determining the risk for obesity and may help develop individually-tailored strategies for obesity prevention.

## Introduction

Variants of the single nucleotide polymorphism (SNP) rs9939609 of fat mass and obesity associated (*FTO)* gene are linked to obesity (Yang et al., 2012; Loos and Yeo, 2014), with the AA and AT genotypes considered at-risk compared to the TT genotype (Frayling et al., 2007; Jacobsson et al., 2012; Sällman Almén et al., 2013). This association, however, seems not to be mediated by dysfunctional metabolism (Cecil et al., 2008; Speakman et al., 2008), but rather stems from increased dietary intake and unhealthy eating behaviors, both in children (Timpson et al., 2008) and adults (Brunkwall et al., 2013). Furthermore, obesity is associated with a wide range of personality traits (Gerlach et al., 2015), such as punishment and reward sensitivity, as measured by the Behavioral Inhibition and Behavioral Activation Scales (BIS and BAS) respectively (Carver and White, 1994). The BIS score positively correlates with inactivity and poor diet, while the BAS negatively correlates with inactivity and poor diet in most of its subscales (Carver and White, 1994; Voigt et al., 2009; Meule, 2013; Dietrich et al., 2014).

While many studies have focused on the influence of the *FTO* variants on food intake and metabolism, few explored their association with cerebral activity. *FTO* is ubiquitously expressed in human tissues, including the brain, where high levels are specifically found in the cerebellum, brainstem nuclei, hypothalamus, and temporal and parietal lobes (Fredriksson et al., 2008; Bressler et al., 2013). Recently, *FTO* variants have been found to modulate the response to food cues in brain regions involved in appetite regulation and reward processing (Karra et al., 2013) as also supported by the observation that carriers of the two variants of the gene respond differently to food stimuli after the ingestion of a glucose solution (Heni et al., 2014). The most prominent differences between those carriers of the at-risk compared to the low-risk alleles were elicited in the prefrontal cortex, a region involved in the inhibitory control of eating, in line with the reported tendency toward loss of control over eating (Tanofsky-Kraff et al., 2009).

To the best of our knowledge, no studies have investigated the link between the *FTO* alleles and the connectivity in the resting-state brain, although a few studies have compared obese to lean participants. In resting-state, participants are not performing a task or responding to external stimuli in the scanner, but simply laying still. In this condition, specific functional networks can be observed across groups (Raichle et al., 2001; Damoiseaux et al., 2006), reflective of the spontaneous mind activity of the participants. Most resting-state studies have focused on the default mode network (DMN; Raichle et al., 2001), the sensorimotor network (SMN; Smith et al., 2009), and the Salience network (SN; Seeley et al., 2007). The DMN, comprising precuneus, posterior cingulate, bilateral inferior–lateral parietal cortices, and ventromedial frontal cortex (Smith et al., 2009), is deactivated during tasks requiring attention (Raichle et al., 2001), while exhibiting intrinsic activity in internally directed cognitive activities (Mason et al., 2007; Raichle and Snyder, 2007; Buckner et al., 2008). The SMN, including supplementary motor area, sensorimotor cortex, and secondary somatosensory cortex (Smith et al., 2009), corresponds to the action–execution programs and perception–somesthesis integration (Smith et al., 2009). The SN, comprising paralimbic structures such as the dorsal anterior cingulate cortex (dACC), the orbital and insular cortices (Seeley et al., 2007), is involved in emotional arousal, reward sensitivity, and decision-making (Seeley et al., 2007).

Alterations in DMN connectivity have been reported in obesity (Kullmann et al., 2012; Paolini et al., 2012)as well as in other eating disorders (McFadden et al., 2014). Resting-state studies of obese participants have demonstrated increased connectivity in the precuneus and decreased connectivity in the right ACC and in the left insula, part of the temporal lobe network (Kullmann et al., 2012). The DMN global efficiency and the capacity to cope with fasting periods have been also found to be predicted by personality traits of the participants, as measured by the Mindfulness Attention Awareness Scale (MAAS), in obesity (Paolini et al., 2012). Within the SN, obese participants have showed increased functional connectivity strength in the putamen at resting-state fMRI (García-García et al., 2013), while decreased connectivity within the SMN has been reported in pre- and post-central gyri (Zhang et al., 2013).

Apart from Zhang et al. (2013), who studied patients with Prader–Willi syndrome, a specific genetic condition associated with hyperfagia and early obesity, none of the previous studies included any genotypization of the participants. Therefore, ours is the first study to focus on the resting-state connectivity in carriers of different alleles of an obesity gene. We focused on the *FTO* gene, particularly the SNP rs9939609, which has been most consistently associated with obesity in different populations (Frayling et al., 2007; Thorleifsson et al., 2009; Willer et al., 2009; Speliotes et al., 2010; Jacobsson et al., 2012; Rosenbloom et al., 2012; Yang et al., 2012; Sällman Almén et al., 2013; Loos and Yeo, 2014; Apalasamy and Mohamed, 2015). Understanding the influence of the *FTO* gene on susceptibility to obesity might help improve current research on prevention strategies to fight obesity based on individual genetics (Razquin et al., 2011). We investigated differences in the resting state networks in participants carrying the non-risk TT or the at-risk AA genotype of the *FTO* SNP rs9939609. Additionally, we examined whether these differences are related to punishment or reward. To this purpose, we calculated the BIS/BAS ratio (BBr) for each participants, which measures a person's predisposition toward an avoidance or approach behavior, respectively reflecting the sensitivity to punishment or rewarding stimuli (Carver and White, 1994). Reward processing and avoidance learning have been found to be modulated by *FTO* variants, and an impaired avoidance learning has been reported in the AA carriers (Sevgi et al., 2015). The BAS has been associated with positive affect, and involves the mesolimbic dopaminergic pathway ascending from the ventral tegmental area to the nucleus accumbens and ventral striatum (Demaree et al., 2005). The BIS, conversely, is associated with negative affect such as fear and anxiety, and is modulated by adrenergic pathways from the locus coeruleus and serotonergic pathways from the raphe nucleus (Demaree et al., 2005). In this framework, the DMN, the SMN and SN were tested, providing clues on the neural systems underlying the tendency toward obesity development.

## Methods

### Participants

Prior to any experimental procedures, all participants gave written informed consent to the study which conformed to the Declaration of Helsinki and was approved by the Ethical Review Board of Uppsala. Participants were 30 right-handed, northern-European males, with a mean age of 26 ± 3 years, recruited locally in Uppsala, Sweden by advertisement. Participants were excluded for claustrophobia, metal implants, psychiatric disorders, smoking, and illicit drug use. Genotyping of the *FTO* single nucleotide polymorphism (SNP) rs9939609 was performed with a pre-designed Taqman single-nucleotide polymorphism genotyping assay (Applied Biosystems, Foster City, USA) and an ABI7900 genetic analyzer with SDS 2.2 software at the Uppsala Genome Center (http://www.genpat.uu.se/node462). The genotype call rate was 97.8%. Only homozygous participants were included in the study. There was a similar distribution of body mass index (BMI) for each genotype of the rs9939609 SNP: the AA genotype had a mean BMI of 26.8 with a range of 13.2 kg/m^2^ (*n* = 13), while the TT genotype had a mean BMI of 24.1 and a range of 9.6 kg/m^2^ (*n* = 17). As BMI does not have a Gaussian distribution in the population (Flegal and Troiano, 2000; Penman and Johnson, 2006; Peeters et al., 2015), we tested this variable for normality using the Shapiro–Wilk's test. Skewness was found to be significant (*p* < 0.01), thus a Mann–Whitney *U*-test was used. The BMI difference between genotypes was found to approach significance (*p* = 0.053).

Clinical measures for punishment sensitivity and reward-seeking behavior were acquired through the Behavioral Inhibition and Activation Systems (BIS and BAS, respectively) questionnaires (Carver and White, 1994). The BIS/BAS scales are composed of 24 items. Each item is represented by a statement, which the participants indicates how much he agrees or disagrees with on a four-point scale. The BIS includes only one scale, evaluating the reactions to the anticipation of punishment, and anxiety. The BAS comprises three subscales: the Drive scale is pertinent to the pursuit of desired goals; the Fun Seeking scale evaluates the desire for new rewards and impulsivity; the Reward Responsiveness scale focuses on the positive reactions anticipating the rewards.

### MRI acquisition

Structural and functional brain images were acquired with a Philips 3-Tesla (Achieva, Philips Healthcare, Best, Netherlands) using a 32-channel head coil. Structural images were acquired first with a T1-weighted turbo-field-echo (TFE) sequence (TR = 8100 ms; TE = 3.7 ms; flip angle: 8°; slice thickness = 1 mm; slice spacing = 1 mm). Structural scans were reviewed by a clinician for abnormalities. 180 resting-state volumes were registered during the T2^*^-weighed echo-planar imaging (EPI) sequence (TR = 2000 ms; TE = 30 ms; flip angle: 90°; slice thickness = 3 mm; slice spacing = 3.9 mm; slices number = 32). The acquisition lasted 6 minutes, although a 4 minutes runlength has been estabilished as sufficient time for reliable estimation of connectivity (Van Dijk et al., 2010). Indeed, the average correlation strengths within the main networks stabilize after 5 min of acquisition, and minimal benefits are observed with extended acquisitions beyond 6 min (Van Dijk et al., 2010).

Participants were scanned in the morning, after an overnight fast and before having any meal. The protocol also included a fMRI session with a block design, prior to the resting-state acquisition. During this session, the participants were shown images of low-calorie food, high-calorie food, or control images in a block design format.

### Pre-processing

Pre-processing was carried out with Data Processing Assistant for Resting-state fMRI Advanced (DPARSFA; http://rfmri.org/) extension in Statistical Parametric Mapping 8 (SPM8; http://www.fil.ion.ucl.ac.uk/spm/software/spm8/ Wellcome Trust Centre for Neuroimaging, University College London) implemented in MATLAB (version r2011a). The first 10 volumes were discarded to allow for signal equilibration. Slice timing was performed and the functional T2 images were realigned to correct for head motion. The structural T1 images were co-registered to the functional images, and the DARTEL (Diffeomorphic Anatomical Registration ThroughExponentiated Lie Algebra; Ashburner, 2007) segmentation option was chosen. The functional images were band-pass filtered for low frequencies fluctuations (hz 0.01–0.08) to reduce the interference of physiological noise, such as respiratory and cardiac artifacts—occurring at higher frequencies (0.3–0.5 Hz; Birn et al., 2006; Van Dijk et al., 2010). Low-frequency fluctuations below 0.1 Hz have been demonstrated to predominate in the cross-correlation coefficients for functionally related regions (Biswal et al., 1995; Cordes et al., 2000; De Luca et al., 2006), with the most consistent correlations occurring within a range of 0.01–0.08 Hz (Van Dijk et al., 2010; Cong et al., 2014). Accordingly, a filter with a low-pass cutoff of 0.08 Hz has been demonstrated to perform better than a low-pass filter of 0.1 Hz (Satterthwaite et al., 2013). The filtered volumes were normalized to the standard anatomical Montreal Neurological Institute (MNI) template (Mazziotta et al., 2001) using 2 × 2 × 2 mm voxel size. Images were smoothed with a 6 full width at half maximum (FWHM) Gaussian kernel to increase the signal to noise ratio and to accommodate for anatomical and functional variability between subjects. The segmented gray matter structural images were imported to MNI space using DARTEL tool implemented in SPM8.

### Independent component analysis

An Independent Component Analysis (ICA) was carried out using the Multivariate Exploratory Linear Optimized Decomposition into Independent Components (MELODIC) software implemented in FSL (*FMRIB* Software Library) package (Jenkinson et al., 2012).

Twenty-one components were identified through a Bayesian dimensionality estimation analysis (Beckmann and Smith, 2004) and visually inspected for network identification. Networks identification was further confirmed by a cross-correlation analysis performed on the brain-maps resting-state networks provided by Smith et al. (2009), and available on the FMRIB's website (http://www.fmrib.ox.ac.uk/analysis/brainmap+rsns/), for the Default Mode Network (DMN) and the Sensorimotor Network (SMN), and on the anterior salience network map provided by Stanford University (http://findlab.stanford.edu/functional_ROIs.html) for the Salience Network (SN). A dual regression analysis was carried out. The resulting connectivity maps were normalized for residual within-subject noise prior to subsequent statistical analysis.

### Statistical analysis

#### Connectivity analysis

Three networks were separately investigated: the DMN, the SMN, and the SN. All statistical analyses were performed using the randomize non-parametric permutation testing (Bullmore et al., 1999) implemented in FSL (*FMRIB* Software Library), applied to the General Linear Model (GLM). A preliminary two groups comparison was performed, testing for group differences between AA and TT participants.The BIS/BAS ratio (BBr) was then calculated (Demaree et al., 2005). The relative strengths of these two systems have indeed been previously used for personality trait assessment (Demaree et al., 2005) and have been related to resting-state activity as measured by electroencephalography (EEG; Sutton and Davidson, 1997; Diego et al., 2001). To this end, the overactivity of one system has been measured either as a BBr (Demaree et al., 2005) or as a normalized BIS-BAS difference (Sutton and Davidson, 1997; Diego et al., 2001). Connectivity was tested for a main effect of BBr and the interaction between the factor genotype and the continuous predictor BBr. When an effect of the BBr or an interaction effect was detected, a *post-hoc* correlation analysis was carried out separately in each group to further investigate the direction of the correlation, and the slopes were tested for significant differences between groups.

We did not include BMI as a covariate because it can be considered as an effect of the eating behavior. Because the BMI difference between groups approached significance, and to further confirm that our findings were not simply due to BMI influence, we also investigated whether a main effect of BMI or an interaction between genotype and BMI existed. To this purpose, the same model and contrasts as specified above were used.

All analyses were performed including demeaned age as a nuisance covariate, and a voxel-based correction was carried out entering the smoothed normalized demeaned gray matter (GM) images as a voxel-dependent regressor, using the—vxl option in FSL. A voxel-based correction was chosen, rather than including the total brain volume as confounding covariate, as specific brain regions are reported to be undergoing a process of progressive atrophy in the carriers of the SNPrs9939609 of *FTO* gene (Ho et al., 2010). The number of permutations was set at 5000 and a threshold-free cluster enhancement (TFCE) approach (Nichols and Holmes, 2002) was used to correct for multiple comparisons. Clusters were considered significant under two conditions: cluster-level *p* < 0.05 after correction for family-wise error (FWE) and a cluster size ≥20 voxels.

Tables and corresponding structures were generated via the Anatomy tool (version 2.0) extension for SPM8. For the structures that could not be identified by the tool, the corresponding regions on the Talairach atlas are reported.

#### Clinical measures

Statistical analysis for BIS score and BAS subscales were performed using Statistical Package for Social Science (SPSS). Distributions were tested for normality and homogeneity of variance prior to testing using the Shapiro–Wilk and Levene's tests respectively. An independent two-samples *t*-test was performed to test for differences between groups with normal distributions and equal variance, while a non-parametric Mann–Whitney test was used if either assumption failed.

The threshold for significance was considered *p* < 0.05. All data are reported as mean ± 1 standard deviation.

## Results

### Greater connectivity in the default mode network for punishment-sensitive participants and differences within each genotype

The DMN is one of the most studied amongst the resting-state networks, and alterations in its connectivity have been reported in obesity (Kullmann et al., 2012; Paolini et al., 2012) as well as in other eating disorders (McFadden et al., 2014).

**Figure 1:**
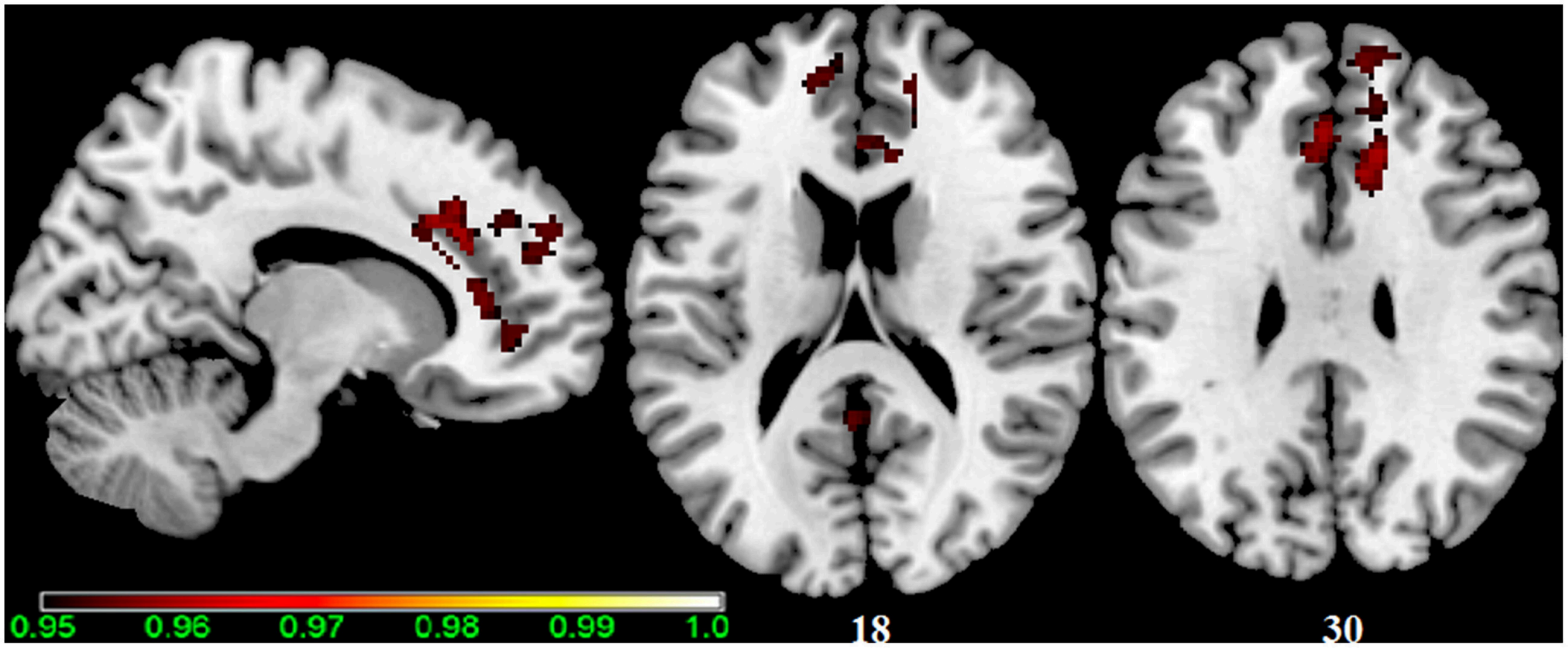
**Main effect of the BBr on Default Mode Network: greater connectivity in punishment sensitive-participants independent of genotype**. Thirty male participants were scanned in a fasted state for resting-state analysis. Participants were split according to their genotype based on the *FTO* SNP rs9939609, and the main effect of the continuous variable BIS/BAS ratio (BBr), a psychometric score for punishment and reward sensitivity, was tested. A high BBr reflected a prominence of the BIS over the BAS. Sagittal and axial slices showing regions where a main effect of BBr on connectivity was detected, corrected for multiple comparison with *p* < 0.05. z-MNI coordinates are reported under axial slices. The colored bar at the bottom left indicates (1−*p*) values.

**Figure 2:**
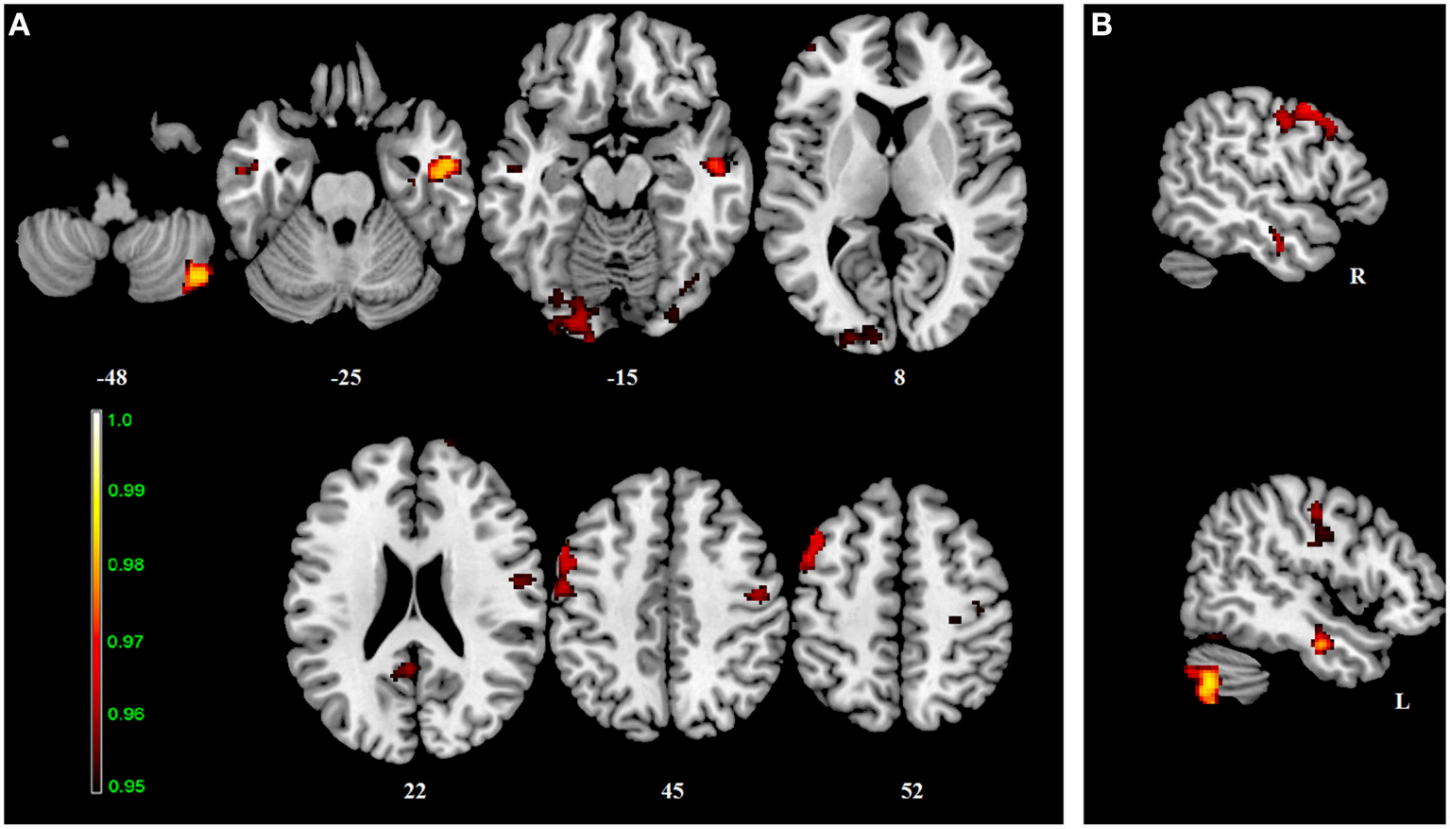
**Interaction effect of the BBr and genotype on Default Mode Network connectivity**. The connectivity was tested for the interaction effect of BBr and genotype. Axial **(A)** and sagittal **(B)** slices showing regions where a significant interaction effect was found, corrected for multiple comparison with *p* < 0.05. z-MNI coordinates are reported under axial slices, and the side is reported under the sagittal slices. The colored bar at the bottom left indicates (1−*p*) values.

No differences in connectivity were detected between the AA and TT genotype in the two groups comparison in our sample. When testing DMN connectivity for effects of BBr and genotype, we found a main effect of BBr in the left superior medial frontal gyrus, the right middle cingulate cortex (MCC) and ACC bilaterally, the right middle orbital gyrus and the precuneus (Figure 1; Table 1). A significant interaction effect between genotype and BBr was also found on connectivity in many temporal regions, such as superior, middle and inferior temporal gyri, parahippocampal gyrus, as well as in the posterior cingulate cortex (PCC), precuneus, frontal areas including the superior and middle frontal gyri and precentral gyrus, and occipital and cerebellar regions (Figure 2; Table 2). At the *post-hoc* analysis, most of these areas showed a higher correlation coefficient between connectivity and BBr in the AA group, compared to the TT group (Table 3). No regions had a stronger correlation in the TT group rather than the AA group.

**Table 1:** **Main effect of the BBr on Default Mode Network connectivity**.

**Cluster**	***p*-value**	**MNI**			**Side**	**Structure**
**Size (voxels)**	**FWE-corr**	***x***	***y***	***z***		
712	0.04	14	28	30	R	Middle cingulate cortex
		10	32	24	R	Anterior cingulate cortex
		12	36	8		
		16	44	10		
		10	22	22		
		16	36	20		
		−2	34	30	L	Anterior cingulate cortex
		−6	24	24		
		10	46	−2	R	Middle orbital gyrus
		12	44	−4
76	0.04	−10	50	20	L	Superior frontal gyrus
		−4	52	22		
		−8	48	14	L	Anterior cingulate cortex
24	0.04	−2	−50	16	L	Precuneus
22	0.05	−14	52	2	L	Superior frontal gyrus
		−8	58	8		
		−8	52	6		

**Table 2:** **Interaction effect between genotype and BBr on Default Mode Network connectivity**.

**Cluster**	***p*-value**	**MNI**			**Side**	**Structure**
**Size (voxels)**	**FWE-corr**	***x***	***y***	***z***		
510	0.01	46	−68	−44	R	Cerebellum (Crus 2)
		42	−70	−48	R	
		50	−70	−40	R	
		40	−68	−50	R	Cerebellum (VII)
		46	−66	−54	R	
		40	−72	−58	R	Inferior Semi-Lunar Lobe
		38	−68	−34	R	Cerebellum (Crus 1)
454	0.03	−50	4	48	L	Precentral gyrus
		−50	8	50		
		−54	10	46		
		−38	2	60		
		−54	12	42		
		−44	20	50	L	Middle frontal gyrus
		−52	18	38		
		−54	−6	42	L	Postcentral gyrus
		−48	−8	38		
		−44	−10	38		
		−40	−12	38		
425	0.02	50	−10	−26	R	Inferior temporal gyrus
		36	−14	−26	R	ParaHippocampal gyrus
		54	−6	−26	R	Middle temporal gyrus
		52	−12	−14		
		68	−16	−12		
		64	−14	−14		
		62	−10	−14		
379	0.04	−20	−92	−14	L	Lingual gyrus
		−14	−102	−14		
		−30	−82	−16		
		−20	−84	−18	L	Fusiform gyrus
		−12	−84	−20	L	Cerebellum (Crus 1)
		−34	−96	−16	L	Inferior occipital gyrus
		−4	−92	−12	L	Calcarine gyrus
364	0.04	46	−12	44	R	Precentral gyrus
		52	−4	22		
		46	−14	28		
		56	−6	18	R	Rolandic operculum
		52	−6	30	R	Postcentral gyrus
		66	−4	14		
239	0.04	−8	−46	26	L	Posterior cingulate cortex
		−4	−50	20		
		−2	−52	18	L	Precuneus
		−12	−56	32		
		−14	−54	30		
209	0.04	−20	−96	8	L	Middle occipital gyrus
		−24	−92	12		
		−24	−88	10		
		−30	−96	−2		
		−34	−96	−4		
		−10	−98	12	L	Superior occipital gyrus
		−8	−96	8		
		−12	−92	8		
70	0.05	32	−98	−12	R	Inferior occipital gyrus
		30	−90	−16		
66	0.04	−52	−8	−26	L	Middle temporal gyrus
		−56	−10	−20		
		−46	−4	−26		
		−52	−14	−14		
65	0.04	42	−66	−20	R	Fusiform gyrus
		36	−76	−16		
		34	−78	−18		
47	0.04	−42	54	10	L	Middle frontal gyrus
33	0.04	−4	60	36	L	Superior medial frontal gyrus
		−8	66	28		
		−6	64	30		
27	0.05	40	−22	62	R	Precentral gyrus

**Table 3:** **Between-group differences in correlations slopes between BBr and connectivity in the Default Mode Network**.

**Cluster**	***p*-value**	**MNI**			**Side**	**Structure**
**Size (voxels)**	**FWE-corr**	***x***	***y***	***z***		
510	0.01	46	−68	−44	R	Cerebellum (Crus 2)
		42	−70	−50		
		50	−70	−40		
		40	−72	−58	R	Cerebellum (Inferior Semi−Lunar Lobe)
		46	−66	−54	R	Cerebellum (VII)
		38	−68	−34	R	Cerebellum (Crus 1)
492	0.03	−50	4	48	L	Precentral gyrus
		−50	8	50		
		−54	10	46		
		−54	12	42		
		−54	−6	42	L	Postcentral gyrus
		−48	−8	38		
		−44	−10	38		
		−40	−12	38		
		−52	18	38	L	Middle frontal gyrus
		−38	4	60		
425	0.01	50	−10	−26	R	Inferior temporal gyrus
		52	−12	−14	R	Middle temporal gyrus
		64	−14	−14		
		62	−10	−14		
		36	−14	−26	R	ParaHippocampal gyrus
414	0.04	46	−12	44	R	Precentral gyrus
		40	−16	52		
		52	−4	22		
		46	−14	28		
		42	−10	30		
		56	−6	18	R	Rolandic operculum
		52	−6	30	R	Postcentral gyrus
		48	−8	34		
		66	−4	14		
379	0.03	−20	−92	−14	L	Lingual gyrus
		−14	−102	−16		
		−30	−82	−16		
		−8	−88	−14		
		−20	−84	−18	L	Fusiform gyrus
		−12	−84	−20	L	Cerebellum (Crus 1)
		−34	−96	−14	L	Inferior occipital gyrus
		−30	−100	−14		
		−4	−92	−12	L	Calcarine gyrus
239	0.04	−6	−48	26	L	Posterior cingulate cortex
		−4	−50	20		
		−2	−52	18	L	Precuneus
		−12	−56	32		
		−14	−54	30		
209	0.04	−20	−96	8	L	Middle occipital gyrus
		−24	−90	10		
		−30	−96	−2		
		−32	−98	−4		
		−10	−98	10	L	Superior occipital gyrus
		−8	−96	8		
		−14	−100	14		
159	0.04	42	−66	−20	R	Fusiform gyrus
		36	−76	−16		
		34	−78	−18		
		32	−98	−12	R	Inferior occipital gyrus
		30	−88	−16		
66	0.03	−52	−8	−26	L	Middle temporal gyrus
		−56	−10	−20		
		−46	−4	−26		
		−52	−14	−14		
51	0.04	−42	54	10	L	Middle frontal gyrus
33	0.04	−4	60	36	L	Superior medial frontal gyrus
		−8	66	28		
28	0.05	40	−22	62	R	Precentral gyrus
		38	−16	62		
23	0.05	28	−26	52	R	Precentral gyrus

**Figure 3:**
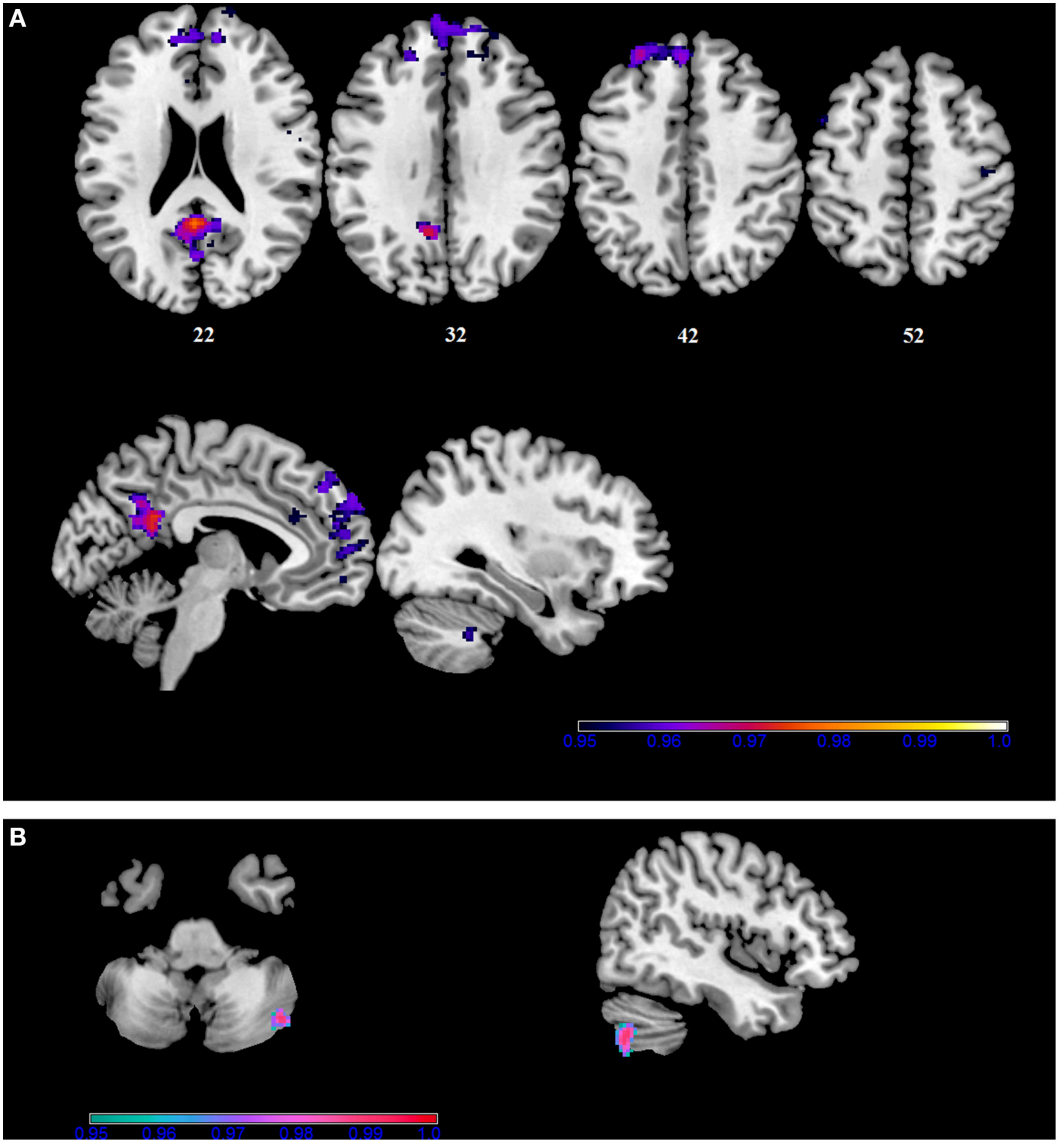
**Within-group correlations between the BBr and Default Mode Network connectivity**. The *post-hoc* analysis was performed separately in each group to test for correlations between the BBr and the connectivity within each group. Axial and sagittal slices showing regions where **(A)** a significant positive correlation was found in the AA group, and **(B)** a negative correlation was found in the TT group, corrected for multiple comparison with *p* < 0.05. z-MNI coordinates are reported under axial slices. The colored bars indicate (1−*p*) values.

Within the AA group, a significant positive correlation was also found between the BBr and connectivity in the superior and middle frontal gyrus, in the left cuneus, precuneus, calcarine gyrus and cingulate cortex, in the right inferior parietal lobule (IPL) and cerebellum (Figure 3; Table 4), while an inverse correlation was found between the BBr and the connectivity in the cerebellum within the TT group (Figure 3; Table 5).

**Table 4:** **Positive correlations between the BBr and Deafult Mode Network connectivity in the AA group**.

**Cluster**	***p*-value**	**MNI**			**Side**	**Structure**
**Size (voxels)**	**FWE-corr**	***x***	***y***	***z***		
997	0.03	−26	46	40	L	Superior frontal gyrus
		−4	42	42	L	Superior medial frontal gyrus
		−2	58	34		
		−8	50	22		
		−2	48	32		
		−4	50	28		
		−10	64	28		
		−6	56	16		
		10	54	30	R	Superior medial frontal gyrus
		10	52	22		
		6	46	38		
641	0.02	−2	−50	18	L	Precuneus
		−4	−62	20		
		−12	−56	24		
		−8	−60	38		
		−6	−48	26	L	Posterior cingulate cortex
		0	−70	26	L	Cuneus
		0	−68	22	L	Calcarine gyrus
		2	−68	12		
166	0.04	−12	52	2	L	Anterior cingulate cortex
		−12	64	6	L	Superior medial frontal gyrus
		−10	66	16		
96	0.03	−40	54	10	L	Middle frontal gyrus
35	0.05	−2	34	30	L	Anterior cingulate cortex
		−4	32	26		
		−4	28	24		
		−4	28	28		
35	0.05	20	66	22	R	Superior Frontal Gyrus
		18	68	18		
		12	68	18	R	Superior medial frontal gyrus
25	0.04	34	−48	−40	R	Inferior parietal lobule
		30	−44	−34	R	Cerebellum (VI)
21	0.04	−48	8	50	L	Precentral Gyrus
21	0.04	26	−40	−44	R	Cerebellum (X)

**Table 5:** **Negative correlations between the BBr and Default Mode Network connectivity in the TT group**.

**Cluster**	***p*-value**	**MNI**			**Side**	**Structure**
**Size (voxels)**	**FWE-corr**	***x***	***y***	***z***		
183	0.01	42	−70	−50	R	Cerebellum (Crus 2)
		46	−68	−44		
		40	−72	−58	R	Cerebellum (Inferior Semi-lunar)

### Greater connectivity in the sensorimotor network for punishment-sensitive AA participants

Alterations in the resting-state connectivity of the SMN have been reported in obese people (Zhang et al., 2013).

**Figure 4:**
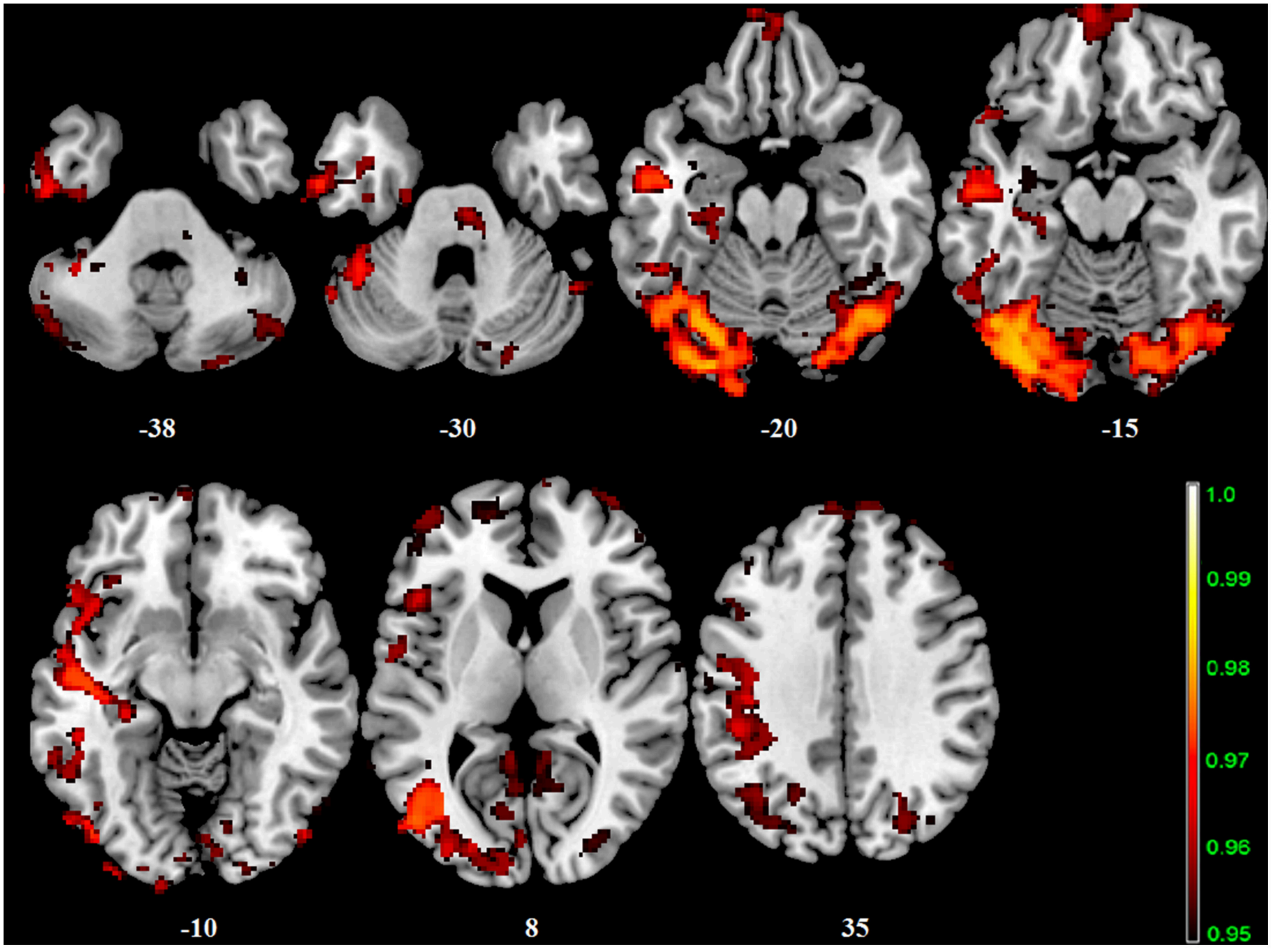
**Interaction effect of the BBr and genotype on SensoriMotor Network connectivity**. The connectivity was tested for the interaction effect of BBr and genotype. Sagittal slices showing regions where a significant interaction effect was found, corrected for multiple comparison with *p* < 0.05. z-MNI coordinates are reported. The colored bar at the bottom right indicates (1−*p*) values.

**Figure 5:**
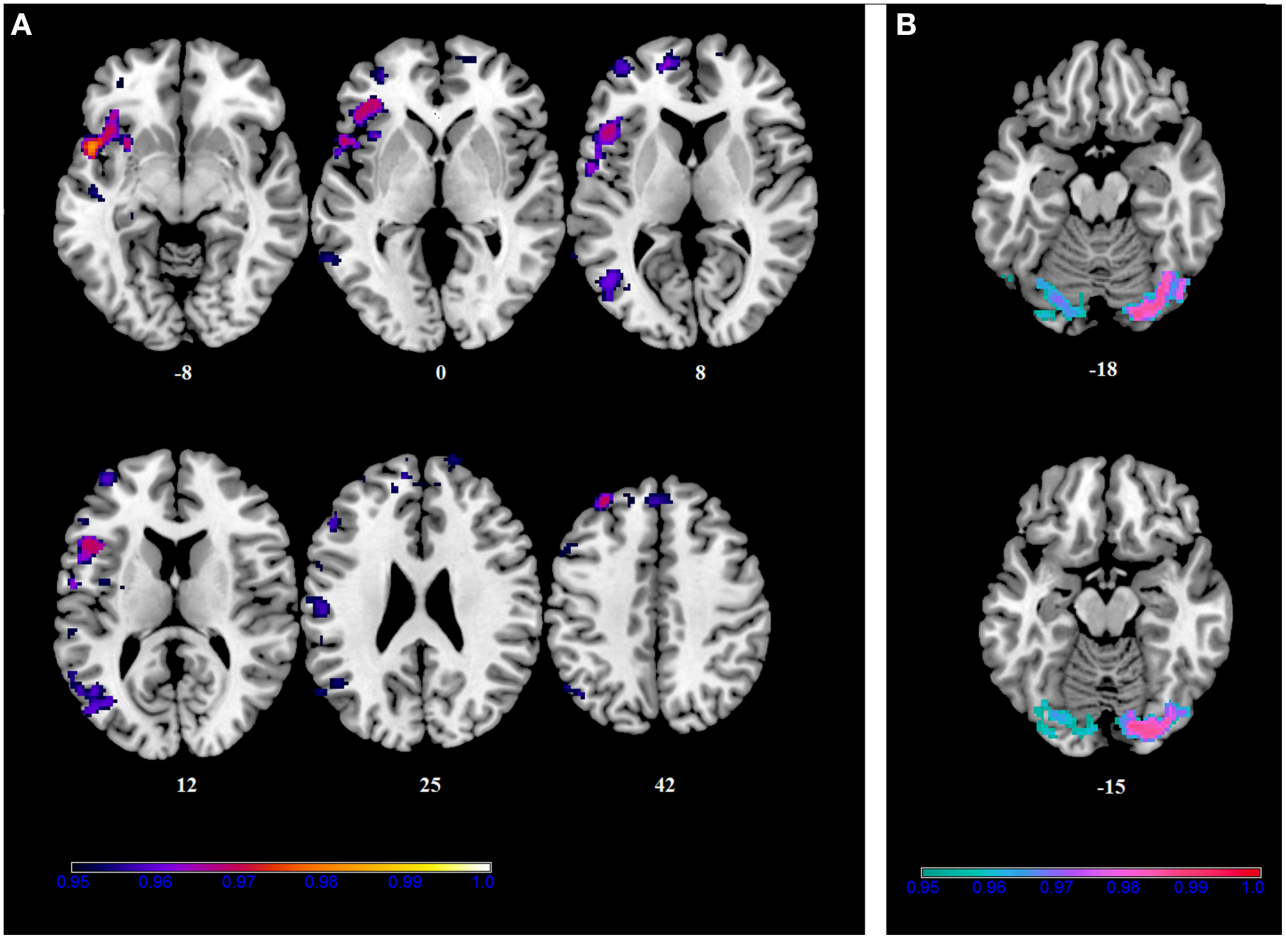
**Within-group correlations between the BBr and SensoriMotor Network connectivity**. The *post-hoc* analysis was performed separately in each group to test for correlations between the BBr and the connectivity within each group. Axial and sagittal slices showing regions where **(A)** a significant positive correlation was found in the AA group, and **(B)** a negative correlation was found in the TT group, corrected for multiple comparison with *p* < 0.05. z-MNI coordinates are reported under axial slices. The colored bars indicate (1−*p*) values.

In our sample, no differences in SMN connectivity were found between genotypes at the two-samples comparison, nor a main effect of the BBr was detected. However, a significant interaction effect was found between genotype and BBr on the connectivity in fronto-temporal regions, including the middle orbital gyrus, the inferior frontal gyrus (IFG), the middle temporal gyrus (MTG) and the insula, as well as the superior parietal lobule (SPL), and occipito-cerebellar areas (Figure 4; Table 6). Most of these regions showed, in the *post-hoc* analysis, a higher correlation coefficient between the BBr and connectivity in the AA group compared to the TT group (Table 7), while the opposite contrast yielded no significant results. Moreover, in the AA participants, the left superior and middle temporal gyri and orbito-frontal regions such as the middle orbital gyrus and superior, medial, and inferior frontal gyri showed a positive correlation with the BBr (Figure 5; Table 8), while in the TT group a negative correlation was found between the BBr and connectivity in occipito-cerebellar regions (Figure 5; Table 9).

**Table 6:** **Interaction effect between BBr and genotype on SensoriMotor Network connectivity**.

**Cluster**	***p*-value**	**MNI**			**Side**	**Structure**
**Size (voxels)**	**FWE-corr**	***x***	***y***	***z***		
5769	0.02	−36	−82	−18	L	Fusiform gyrus
		−36	−72	−16		
		−30	−88	−18	L	Lingual gyrus
		−18	−90	−18		
		−24	−76	−20	L	Cerebellum (VI)
		42	−66	−20	R	Fusiform gyrus
		34	−74	−20	R	Cerebellum (VI)
		18	−84	−16		
		36	−80	−18	R	Lingual gyrus
		16	−78	−14	R	Lingual gyrus
		−50	−68	−24	L	Cerebellum (Crus 1)
4055	0.02	−46	−6	−24	L	Middle temporal gyrus
		−46	−16	−10		
		−50	−14	−14		
		−48	−2	−50		
		−60	−12	−26	L	Inferior temporal gyrus
		−58	−10	−32		
		−50	−12	−38		
		−52	−4	−44		
		−52	2	−44		
		−38	−22	−10	R	Insula
		−42	−24	40	L	Postcentral gyrus
1125	0.03	−46	20	10	L	Inferior frontal gyrus (p. Triangularis)
		−42	18	12		
		−36	34	14		
		−50	30	24		
		−48	34	24		
		−46	54	8	L	Middle temporal gyrus
		−16	58	6	L	Superior frontal gyrus
		−42	58	6	L	Middle frontal gyrus
		−34	56	22		
		−36	56	18		
		−44	34	30		
1029	0.03	24	−60	48	R	Superior parietal lobule
		18	−64	50		
		26	−76	52		
		22	−66	60		
		24	−76	58		
		30	−42	68	R	Postcentral gyrus
		40	−38	60		
		20	−62	48	L	Precuneus
		12	−66	52	R	Precuneus
		24	−66	36	R	Superior occipital gyrus
		26	−74	34		
980	0.03	40	56	16	R	Middle frontal gyrus
		32	54	30		
		48	40	24		
		48	48	16		
		38	64	8		
		36	62	4		
		50	44	26		
		32	66	10		
		−6	62	32	L	Superior medial frontal gyrus
		18	68	20	R	Superior medial frontal gyrus
		12	60	36		
639	0.04	−26	12	64	L	Middle frontal gyrus
		−52	16	44		
		−44	20	50		
		−46	12	52		
		−44	16	52		
		−24	6	68	L	Superior frontal gyrus
		−54	12	44	L	Precentral gyrus
		−56	10	40		
		−48	10	30		
		−54	2	44		
		−52	4	46		
528	0.04	−6	−48	8	L	Precuneus
		0	−54	18		
		2	−52	20	R	Precuneus
		8	−48	8		
		10	−46	6		
		12	−44	2	R	Lingual gyrus
		14	−60	8		
		4	−62	8		
		−12	−54	30	L	Precuneus
		6	−64	10	R	Calcarine gyrus
		4	−68	12		
484	0.04	−38	−58	32	L	Angular gyrus
		−42	−70	32		
		−22	−64	34	L	Middle occipital gyrus
		−30	−78	34		
		−24	−80	32	L	Superior occipital gyrus
		−18	−66	40		
295	0.03	−6	66	−18	L	Medial frontal gyrus
		−4	62	−16	L	Rectal gyrus
		0	56	−20		
		2	60	−20	R	Superior frontal gyrus
		10	68	−16	R	Middle orbital gyrus
		−14	64	−14	L	Superior orbital gyrus
270	0.03	46	−68	−52	R	Cerebellum (Crus 2)
		50	−70	−40		
		42	−74	−40		
		38	−68	−34	R	Cerebellum (Crus 1)
157	0.03	10	−28	−32	R	Cerebellum (Culmen)
		8	−22	−32		N/A
		4	−22	−28		N/A
		2	−26	−32		N/A
		0	−28	−34		N/A
100	0.04	28	−86	10	R	Middle occipital gyrus
		22	−92	14	R	Superior occipital gyrus
92	0.04	−58	−34	16	L	Superior temporal gyrus
89	0.04	66	−14	24	R	Postcentral gyrus
		68	−16	18		
		66	−8	18		
85	0.04	−64	−52	2	L	Middle temporal gyrus
59	0.05	−54	−26	2	L	Superior temporal gyrus
		−60	−28	2	L	Middle temporal gyrus
		−56	−32	−2		
47	0.05	−32	−10	−16	L	Hippocampus
		−30	−6	−18		
		−34	−6	−16	R	ParaHippocampal gyrus
46	0.05	38	−52	−22	R	Fusiform gyrus
		44	−48	−22		
35	0.04	24	−42	−44	R	Cerebellum (X)
		28	−42	−50	R	Cerebellum (VIII)
33	0.04	−36	36	0	L	Inferior frontal gyrus (p. Triangularis)
21	0.05	34	−48	−42	R	Cerebellum (Tonsil)
22	0.05	56	−30	52	R	Inferior parietal lobule
		56	−34	50	R	SupraMarginal gyrus

**Table 7:** **Between-group differences in correlations slopes between BBr and connectivity in the SensoriMotor Network**.

**Cluster**	***p*-value**	**MNI**			**Side**	**Structure**
**Size (voxels)**	**FWE-corr**	***x***	***y***	***z***		
3677	0.02	−30	−88	−18	L	Lingual gyrus
		−20	−90	−18		
		−16	−88	−18		
		−36	−82	−18	L	Fusiform gyrus
		−44	−64	−18		
		−42	−60	−20		
		−48	−64	−20		
		−30	−70	−20	L	Cerebellum (VI)
		−26	−74	−20		
		−46	−70	−16	L	Inferior occipital gyrus
		−18	−84	−20	L	Cerebellum (Crus 1)
3292	0.03	−46	−6	−24	L	Middle temporal gyrus
		−56	−12	−18		
		−64	−8	−26		
		−56	−4	−16		
		−48	−2	−50		
		−34	−24	−8	L	Caudate
		−28	−30	−8	L	Hippocampus
		−38	−60	34	L	Angular gyrus
		−62	−12	−26	L	Inferior temporal gyrus
		−58	−10	−32		
		−40	2	−48		
969	0.02	42	−66	−20	R	Fusiform gyrus
		36	−72	−20		
		48	−70	−18		
		32	−74	−20	R	Cerebellum (VI)
		30	−76	−22		
		18	−84	−16		
		16	−78	−14	R	Lingual gyrus
		24	−82	−22	R	Cerebellum (Crus 1)
		22	−88	−18		
		26	−84	−18		
		52	−74	−16	R	Middle occipital gyrus
515	0.04	38	−44	70	R	Postcentral gyrus
		24	−42	72		
		30	−46	74		
		40	−38	60		
		44	−36	58		
		42	−48	64	R	Superior parietal lobule
		38	−46	62		
		22	−70	54		
		20	−64	50		
		24	−60	48		
		12	−66	52	R	Precuneus
238	0.03	44	−68	−52	R	Cerebellum (Crus 2)
		42	−74	−40		
		38	−68	−36	R	Cerebellum (Crus 1)
221	0.04	42	56	16	R	Middle frontal gyrus
		50	40	24		
		48	48	16		
		36	62	4		
		32	66	10		
718	0.04	−52	14	44	L	Middle frontal gyrus
		−46	20	48		
		−44	12	52		
		−50	10	48	L	Precentral gyrus
107	0.04	10	−28	−32	R	Cerebellum (Culmen)
		4	−22	−28		N/A
		−2	−22	−28		N/A
		0	−28	−34		N/A
96	0.04	−30	10	62	L	Middle frontal gyrus
71	0.04	−46	20	10	L	Inferior frontal gyrus (p. Triangularis)
		−42	18	12		
67	0.05	−6	−48	10	L	Precuneus
		0	−54	16		
		−2	−50	14		
		−2	−50	20	L	Posterior cingulate cortex
50	0.05	28	54	32	R	Middle frontal gyrus
		32	54	30		
45	0.05	18	66	24	R	Superior frontal gyrus
		16	62	30		
44	0.04	−58	−34	16	L	Superior temporal gyrus
32	0.05	−42	60	6	L	Middle frontal gyrus
		−40	56	10		
		−44	54	8		
27	0.04	−64	−52	4	L	Middle temporal gyrus
25	0.04	−6	66	−18	L	Medial frontal gyrus
21	0.04	−30	44	42	L	Superior frontal gyrus

**Table 8:** **Positive correlations between BBr and SensoriMotor Network connectivity in the AA group**.

**Cluster**	***p*-value**	**MNI**			**Side**	**Structure**
**Size (voxels)**	**FWE-corr**	***x***	***y***	***z***		
1264	0.02	−50	10	−8	L	Temporal Pole
		−32	10	−6	L	Claustrum
		−44	16	−10	L	Inferior frontal gyrus (p. Orbitalis)
		−42	24	−4		
		−38	28	−8		
		−48	18	10	L	Inferior frontal gyrus (p. Opercularis)
		−54	10	16		
		−50	12	4		
		−44	30	0	L	Inferior frontal gyrus (p. Triangularis)
		−38	34	0		
		−56	−2	6	L	Rolandic operculum
799	0.04	−46	−66	8	L	Middle temporal gyrus
		−54	−64	12		
		−58	−60	14		
		−58	−54	10		
		−44	−64	34	L	Angular gyrus
		−50	−64	30		
		−56	−56	34		
		−48	−76	12	L	Middle occipital gyrus
		−38	−72	16		
		−36	−70	10		
		−48	−58	48	L	Inferior parietal lobule
757	0.04	−32	50	2	L	Middle frontal gyrus
		−38	52	4		
		−36	54	16		
		−24	54	28		
		−32	56	26		
		−14	50	20	L	Superior frontal gyrus
		0	42	40	L	Superior medial frontal gyrus
		2	56	36		
		30	54	30	R	Middle frontal gyrus
		6	46	38	R	Superior medial frontal gyrus
		20	66	24	R	Superior frontal gyrus
163	0.04	−44	12	52	L	Middle frontal gyrus
		−50	18	40		
		−46	18	40		
		−36	4	60		
		−50	12	48		
		−52	14	46		
131	0.04	−56	−20	24	L	Postcentral gyrus
		−64	−14	24		
		−66	−16	26		
		−48	−16	28		
106	0.04	−16	58	6	L	Superior frontal gyrus
93	0.04	−26	14	64	L	Middle frontal gyrus
		−30	10	66		
92	0.04	−64	−52	4	L	Middle temporal gyrus
		−60	−54	2		
		−60	−46	−2		
85	0.04	−44	−18	−10	L	Superior temporal gyrus
		−38	−22	−16	L	ParaHippocampal gyrus
64	0.03	−30	42	42	L	Superior frontal gyrus
57	0.05	−58	−34	16	L	Superior temporal gyrus
		−56	−36	24	L	Supramarginal Gyrus
57	0.04	−46	−2	−50	L	Inferior temporal gyrus
		−52	2	−44		
		−52	−4	−44		
54	0.04	−30	−6	−18	L	Hippocampus
		−34	0	−18	L	Superior temporal gyrus
44	0.05	−46	−6	−24	L	Middle temporal gyrus
		−54	−6	−24		
		−50	−4	−16		
30	0.05	−14	44	44	L	Superior frontal gyrus
		−16	40	40		
25	0.05	−36	−82	−18	L	Fusiform gyrus
20	0.05	10	70	16	R	Superior medial frontal gyrus
18	0.05	20	58	0	R	Superior frontal gyrus
		14	58	−2	R	Middle orbital gyrus

**Table 9:** **Negative correlations between BBr and SensoriMotor network connectivity in the TT group**.

**Cluster**	***p*-value**	**MNI**			**Side**	**Structure**
**Size (voxels)**	**FWE-corr**	***x***	***y***	***z***		
525	0.01	14	−84	−14	R	Lingual gyrus
		8	−82	−12		
		16	−78	−14		
		44	−78	−18		
		22	−88	−18	R	Cerebelum (Crus 1)
		22	−82	−22		
		42	−66	−20	R	Fusiform gyrus
		28	−84	−18		
212	0.03	−24	−78	−18	L	Fusiform gyrus
		−36	−70	−14		
		−38	−76	−16		
		−18	−86	−18	L	Cerebelum (Crus 1)
		−30	−72	−20	L	Cerebelum (VI)
		−10	−76	−18		
		−30	−88	−18	L	Lingual gyrus
		−8	−86	−16		

### Greater connectivity in the salience network in punishment-sensitive participants and differences within each genotype

The SN comprises paralimbic structures related to reward sensitivity and the elaboration of behavioral responses based on the attribution of value to salient stimuli (Lamichhane and Dhamala, 2015). Reward sensitivity anomalies have been traditionally linked to obesity (Gerlach et al., 2015) and accordingly SN connectivity alterations have been found in obese participants during resting-state (García-García et al., 2013).

**Figure 6:**
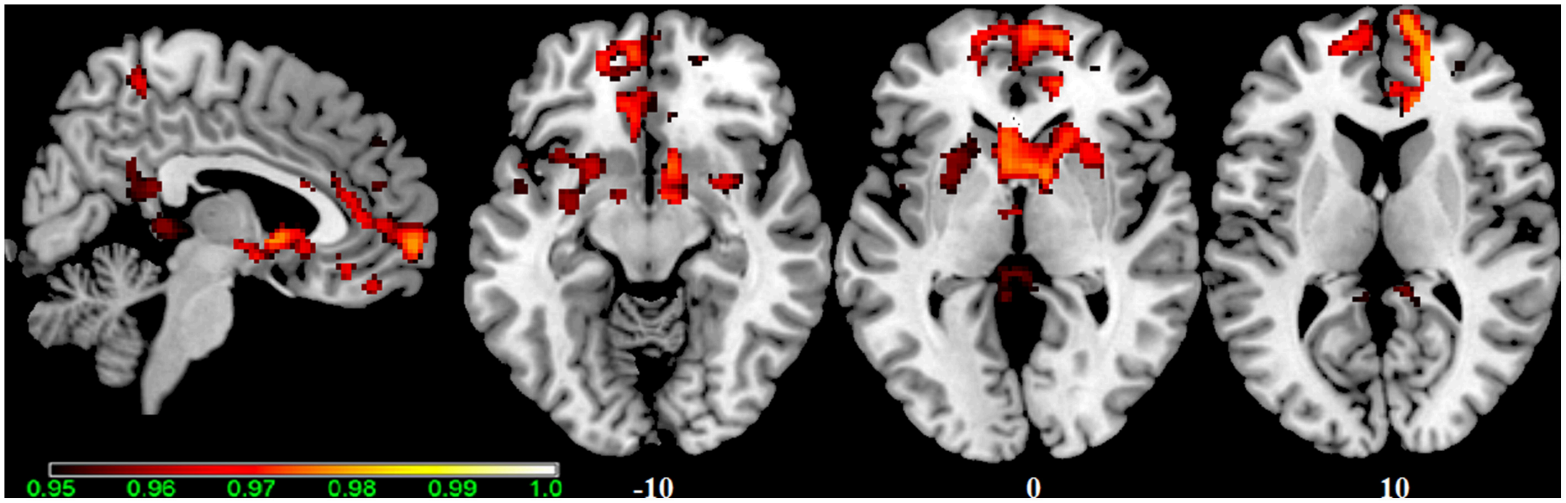
**Main effect of the BBr on Salience Network: greater connectivity in punishment sensitive-participants independent of genotype**. Connectivity was tested for the main effect of the continuous variable BBr. Sagittal and axial slices showing regions where a main effect of BBr on connectivity was detected, corrected for multiple comparison with *p* < 0.05. z-MNI coordinates are reported under axial slices. The colored bar at the bottom left indicates (1−*p*) values.

**Figure 7:**
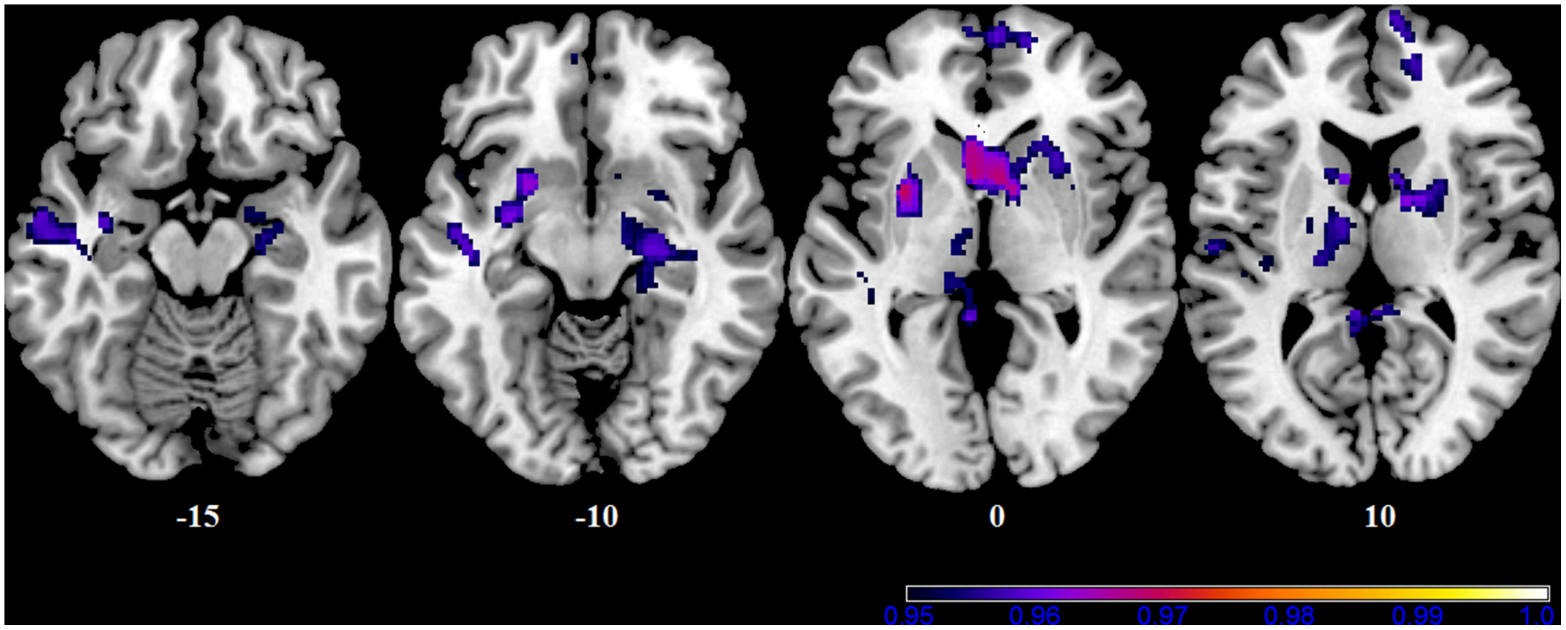
**Correlation between the BBr and Salience Network connectivity within the AA group**. The *post-hoc* analysis was performed separately in each group to test for correlations between the BBr and the connectivity within each group. Axial showing regions where a significant positive correlation was found in the AA group, corrected for multiple comparison with *p* < 0.05. z-MNI coordinates are reported under axial slices. The colored bars indicate (1−*p*) values. No significant correlations were found in the TT group.

In our cohort, no differences in SN connectivity were found between genotypes at the two-samples comparison. A main effect of BBr was detected on the connectivity within the SN in the right ACC, MCC, and PCC, in the right superior frontal gyrus extending to the right middle orbital gyrus, in the caudate and precuneus bilaterally, in the right postcentral gyrus, in the right SPL and in the right paracentral gyrus (Figure 6; Table 10). Moreover, an effect of the interaction between the genotype and the BBr was found on the connectivity in the left MTG (p FWE-corr < 0.05). At the *post-hoc* analysis, indeed, the correlation between the connectivity in the left MTG and the BBr was found to be significantly stronger in the AA group than in the TT group (p FWE-corr < 0.04). Within the AA genotype a positive correlation was also found between the BBr and the connectivity in the left thalamus and temporal areas including the insula and Heschl's gyrus and in the rolandic operculum, as well as in the right hippocampus, in the PCC, deep gray matter nuclei such as the putamen and caudate, and orbito-frontal regions bilaterally (Figure 7; Table 11). No correlations were found in the TT group between connectivity and BBr.

**Table 10:** **Main effect of the BBr on Salience Network connectivity**.

**Cluster**	***p*-value**	**MNI**			**Side**	**Structure**
**Size (voxels)**	**FWE-corr**	***x***	***y***	***z***		
4497	0.02	18	46	8	R	Anterior cingulate cortex
		16	58	10	R	Superior frontal gyrus
		14	56	18		
		12	64	10		
		16	58	−2		
		−6	22	20	L	Anterior cingulate cortex
		4	62	−4	R	Middle orbital gyrus
		−4	54	−8	L	Middle orbital gyrus
		6	−2	−6	R	Hypothalamus
		−4	8	−2	L	Caudate nucleus
		8	10	−10	R	Caudate nucleus
907	0.03	8	−44	56	R	Precuneus
		8	−48	62		
		−14	−48	48	L	Precuneus
		28	−46	64	R	Postcentral gyrus
		24	−42	66		
		18	−44	62		
		28	−42	58		
		32	−52	62	R	Superior parietal lobule
		34	−50	64		
		−6	−44	52	L	Middle cingulate cortex
		10	−34	48	R	Paracentral lobule
506	0.04	2	−42	18	R	Posterior cingulate cortex
		10	−38	8		
		4	−48	28		
		−8	−42	14	L	Posterior cingulate cortex
		−6	−38	20		
		4	−36	4	R	Thalamus
		−2	−32	4	L	Thalamus
		0	−30	2		
		−4	−42	2	L	Cerebellum (Culmen)
		8	−50	22	R	Precuneus
		−6	−42	−4	L	Cerebellum (IV-V)
227	0.04	−10	50	22	L	Superior frontal gyrus
		−2	52	22		
		−12	44	22		
		−8	38	20	L	Anterior cingulate cortex
80	0.05	16	24	30	R	Middle cingulate cortex
		8	44	34	R	Superior frontal gyrus
		14	36	30	R	Medial frontal gyrus
30	0.05	30	48	6	R	Middle frontal gyrus
		28	48	0		
23	0.04	−18	−18	24	L	Caudate nucleus

**Table 11:** **Positive correlations between the BBr and Salience Network connectivity in the AA group**.

**Cluster**	***p*-value**	**MNI**			**Side**	**Structure**
**Size (voxels)**	**FWE-corr**	***x***	***y***	***z***		
1235	0.03	−4	14	2	L	Caudate nucleus
		−8	10	10		
		4	10	0	R	Caudate nucleus
		10	6	0		
		22	8	18		
		12	8	10		
		20	2	12	R	Putamen
		26	16	0		
		24	−16	−14	R	Hippocampus
		22	−24	−12		
		10	−2	−8	R	Pallidum
655	0.03	−4	−42	4	L	Posterior cingulate cortex [-9pt]
		0	−48	14		
		2	−48	30		
		−4	−38	20		
		−2	−32	4	L	Thalamus
		−12	−30	0		
		−8	−30	0		
		−10	−26	−4		
		−6	−34	0		
		6	−50	22	R	Precuneus
		−6	−24	−6	L	Red Nucleus
488	0.04	−56	−10	−20	L	Middle temporal gyrus
		−58	−4	−16		
		−48	−4	−26		
		−48	−12	−12	L	Superior temporal gyrus
[-9pt]		−56	−16	12		
		−50	−8	−6		
		−52	−26	4		
		−44	−18	−10		
		−42	−34	0		
		−50	−26	14	L	Rolandic operculum
		−42	−30	−4	L	Insula
392	0.03	−30	4	0	L	Putamen
		−24	8	−8		
		−20	16	−4		
		−22	−10	8		
		−22	6	−12	L	Subcallosal gyrus
		−34	−8	−16	L	ParaHippocampal gyrus
		−20	−6	6	L	Pallidum
224	0.04	−20	−16	22	L	Caudate nucleus
		−14	−8	12	L	Thalamus
		−16	−10	16		
		−10	−8	10		
		−18	−20	10		
		−14	−16	8		
		−12	−18	0		
		−8	−12	−2		
171	0.04	4	62	−4	R	Middle orbital gyrus
		12	62	−2		
		16	58	−2	R	Superior medial frontal gyrus
		−6	62	−4	L	Middle orbital gyrus
		−4	54	−8		
90	0.04	10	68	10	R	Superior medial frontal gyrus
		8	68	18		
		0	68	16		
82	0.05	−14	−54	30	L	Precuneus
		−10	−62	38		
80	0.04	18	48	8	R	Medial frontal gyrus
		16	52	10	R	Superior medial frontal gyrus
		14	54	18		
24	0.05	−18	54	6	L	Superior frontal gyrus
		−16	62	4		
23	0.05	−38	−22	8	L	Heschls gyrus

### No detectable influence of body mass index on connectivity

To confirm that these findings were not due to a BMI influence, we also investigated the connectivity for a main effect of BMI and an interaction between genotype and BMI. No effect was detected.

### Clinical measures

No differences were found between the AA and TT groups in the BIS and BAS scores. The clinical data of the different groups are found in Table 12.

**Table 12:** **Psychological scores in at-risk and non-risk FTO allele carriers**.

	**AA group (mean, sd)**	**TT group (mean, sd)**
Age	25.8 ± 3.4	25.7 ± 2.1
BMI	26.8 ± 4.3	24.1 ± 2.8
BIS score	18 ± 3	18 ± 4
BAS score	37 ± 5	37 ± 6
Drive	9 ± 2	8 ± 3
Reward Responsiveness	15 ± 2	16 ± 2
Fun Seeking	12 ± 3	12 ± 3

## Discussion

We investigated whether differences existed in resting-state connectivity, within the default mode network (DMN), the sensorimotor network (SMN), and the salience network (SN), between the *FTO* SNP rs9939609 “at-risk” AA genotype and the “non-risk” TT genotype carriers. To gain further insight into the mechanisms underlying the different behaviors observed in the two genotypes, we also investigated correlations between the connectivity in these three networks and the ratio on the Behavioral Inhibition System (BIS), which is associated with behavioral withdrawal, punishment and unhealthy behavior (Carver and White, 1994; Voigt et al., 2009), and the Behavioral Activation System (BAS), comprised of subscales reflecting impulsivity, approach behaviors and reward sensitivity (Carver and White, 1994).

We found that the participants characterized by an imbalance toward BIS prominence (i.e. higher BIS/BAS ratio, BBr) had greater connectivity in many DMN, SMN and SN areas, but the patterns of involvement differed in the two genotype groups. To confirm that these findings were not due to a body mass index (BMI) influence, we also investigated the connectivity of the DMN, SMN, and SN for a main effect of BMI. No effect was detected in any of these networks.

Many structures in the DMN were affected by the BBr, such as the precuneus, the cingulate cortex (CC) and the frontal areas. In each of these, the connectivity increased for higher BBr. These regions are the core of social cognition, and include a wide range of functions, such as self-reflection and self-perception (Amodio and Frith, 2006), with the precuneus also playing a role in self-reference and rumination, and the ACC strongly involved in emotional arousal (Gusnard et al., 2001; Gröne et al., 2015). The orbito-frontal cortex has been also specifically associated with the encoding of food reward and regulation of food behavior, related to both internal and external perceptions (Karra et al., 2013). The connectivity in the para-hippocampus also increased with the BBr. This result is perhaps expected, as the participants were fasting and the activity in this region inversely correlates to the feeling of satiety (Leidy et al., 2011).

Between genotypes, however, the influence of the BBr on the connectivity within the DMN was remarkably different. Within the AA genotype, as BBr increased the connectivity was stronger in frontal and occipital cortices, the PCC and ACC, the IPL and in the cerebellum. Interestingly, in the TT genotype, the connectivity in the cerebellum had a significant inverse correlation with the BBr, opposite to the AA genotype.

The relationship between BBr and connectivity, indeed, was significantly stronger in the AA group not only in the cerebellum, but also in the precentral and postcentral gyri, in the superior and middle frontal gyrus, in the auditory cortex (inferior and middle temporal gyri), in the parahippocampal gyrus, in the PCC, precuneus and occipital areas including lingual gyrus, and visual cortex. The lingual gyrus is involved in the processing of complex visual stimuli (Brunet et al., 2000), which have been observed to be differently handled by the carriers of the two alleles of the *FTO* gene in the fasting state (Karra et al., 2013). The connectivity between the insula/auditory cortex and sensorimotor and visual cortices has been observed to increase in a fasting condition during the resting state if the subject had been previously shown food cues (Paolini et al., 2012). This is also the case of our sample, as the resting state acquisition followed an fMRI protocol recording blood oxygen level dependent (BOLD) activity during the presentation of food images. Moreover, the activity in the somatosensory cortex, located in the postcentral gyrus, in the cerebellum, regulated by the activation of the dopamine type-2/3 (D2/D3) striatal receptors, and in the premotor areas, has also been reported to increase after the presentation of food cues, and to be related to BMI (Tomasi et al., 2015). The cerebellum has also been associated with reward-based learning (Thoma et al., 2008) and the regulation of visceral functions and feeding control, as well as to taste perception in hunger state along with the insular cortex (Tomasi et al., 2015), suggesting that the AA participants attribute a greater reward value to food.

The connectivity in the SMN was also found to be related to the genotype and the BBr, showing a significantly stronger increase for higher BBr in the AA group, rather than in the TT group, in the occipito-cerebellar cortices, in temporal areas including the insular cortex, and in fronto-orbital regions including the superior, middle and inferior frontal gyri and the middle orbital gyrus. Remarkably, the anterior insula and the IFG, together with the orbito-frontal cortex are part of a dopaminergic cortico-striatal circuit involved in inhibitory control, decision making, emotional regulation, motivation, and salience attribution (Tomasi et al., 2015), a finding of particular interest as the *FTO* obesity-associated SNPs correlate with emotional and uncontrolled eating (Cornelis et al., 2014). Furthermore, the IFG and the insula connectivity have been associated with the perception of visceral states (Molnar-Szakacs and Uddin, 2013).

In particular, in the AA group, the connectivity in the fronto-orbital cortex, including IFG, middle and superior frontal gyri, and temporal regions, increased with higher BBr, while in the TT group a negative correlation was found with the connectivity in cerebellar-occipital regions.

Differences in connectivity according to both BBr and genotype were also found in the SN. The SN comprises most of the paralimbic structures. Its core regions, namely the dorsal ACC and anterior insula, are involved in interoceptive-autonomic processing and are co-activated in response to metabolic stress and hunger (Craig, 2002, 2009), while most of the remaining nodes have been associated with the processing of emotions, reward, and homeostatic regulation (Seeley et al., 2007). Reward sensitivity anomalies have been traditionally linked to obesity (Gerlach et al., 2015) and accordingly SN connectivity alterations have been found in obese participants both during resting-state (García-García et al., 2013) and task-related conditions (Kullmann et al., 2013). In our cohort, SN connectivity was greater for higher BBr regardless of genotype, specifically in the precuneus, in the caudate, in the SPL, postcentral and paracentral gyri, in the ACC and PCC bilaterally, in the hypothalamus, and the orbito-frontal regions. The arcuate nucleus of the hypothalamus is influenced by energy intake and glucose circulating levels (Harbron et al., 2014), while the paracentral lobule is part of the reward system and has been shown to activate in response to highly rewarding stimuli (Stice and Yokum, 2014).

Interestingly, in the AA group, but not in the TT group, the connectivity increased with the BBr in the left rolandic operculum, insula, superior temporal gyrus (STG) and Heschls gyrus, as well as in the right hippocampus and parahippocampal gyrus and bilaterally in fronto-orbital regions. Of particular interest is the relationship between BBr and connectivity in a sub-network encompassing the parahippocampal and subcallosal cortices (olfactory and paraolfactory areas), the insula, the rolandic operculum, and the STG for the AA genotype but not the TT genotype. The insular cortex is suggested to be the hub where integration occurs between food smells and food taste, perceived respectively by the parahippocampal/subcallosal and opercularis cortices (Seubert et al., 2015). The connectivity between these regions and temporal areas involved in olfactory memory has also been related to food preferences and the consumption of fatty foods (Weltens et al., 2014). Moreover, hypersensitivity of the somatosensory rolandic operculum has been related to BMI increase (Ng et al., 2011). In contrast with our finding, the resting-state connectivity of the insular cortex has been reported to be decreased in the general obese population (Kullmann et al., 2012), suggesting different responses to food cues may be mediated by other factors, such as genotype.

The nigrostriatal dopaminergic circuitry was also found to be related to the BBr in the AA group. In particular, the connectivity in the putamen, caudate, pallidum, and thalamus increased with higher BBr. Interestingly, the BAS is related to the mesolimbic dopaminergic pathway ascending from the ventral tegmental area to the nucleus accumbens and ventral striatum (Demaree et al., 2005), involved in reward-based processing and avoidance learning, and impaired in carriers of the AA genotype (Sevgi et al., 2015). In particular, connectivity in this circuitry is unexpectedly higher in participants performing worse on avoidance learning tasks (Sevgi et al., 2015). Thus, impairment in avoidance-learning might be related to D2 receptor density in the striatum, which indeed, is lower in the carriers of the A allele (Pohjalainen et al., 1998; Jönsson et al., 1999; Frank et al., 2007). Our findings, showing greater connectivity in this circuitry in AA carriers with lower BAS scores, support this hypothesis.

In all the three networks, connectivity in the MTG showed a significantly different relationship with the BBr between groups, with a greater increase in the AA group. The MTG is involved in addiction, and its engagement occurs in response to both drug cues (Jasinska et al., 2014) and stress imagery (Hommer et al., 2013). Regarding these recent findings, two aspects of the relationship between the BBr and connectivity in our sample are worth noticing: first, as above mentioned, our participants had been shown food cues prior to the resting-state acquisition, and similarities in the activation pattern following drug cues and food cues has been vastly reported (Kelley et al., 2005; Volkow and Wise, 2005; Tang et al., 2012); secondly, the relationship between MTG connectivity and imbalance toward the BIS (Demaree et al., 2005) and poor diet and inactivity (Carver and White, 1994; Voigt et al., 2009; Meule, 2013; Dietrich et al., 2014) was stronger in the AA genotype. Indeed, this genotype confers a predisposition to unhealthy behaviors (Brunkwall et al., 2013). Thus, the AA genotype might play a role in determining an imbalance toward the BIS. This suggests that neural connectivity patterns might more closely influence the sensitivity toward punishment and reward in the AA carriers, affecting reward processing and avoidance learning. Social and environmental factors might consequently act on this predisposed neural substrate with greater impact, increasing the risk of developing obesity.

Given the novelty of our work, some limitations are to be considered. First of all, the small sample size allowed us to perform an exploratory analysis, however further studies will be necessary to confirm these preliminary results. The homogeneity of our sample, including only Swedish males, also calls for the assessment of reproducibility in different populations, as differences in the BIS/BAS questionnaires score have been reported to exist between males and females and possibly between different nationalities (Carver and White, 1994; Dietrich et al., 2014). Furthermore, we scanned the participants in the fasted state, which is known to alter resting-state connectivity (Wijngaarden et al., 2015; Zhang et al., 2015) and after the presentation of food cues. Thus, further studies are needed to clarify whether the alterations we assessed are still detectable in carriers of different allele of the *FTO* gene in different scanning conditions.

## Conclusions

For the DMN, SMN, and SN connectivity, a main effect of the BBr was observed, with connectivity between frontal, temporal, and paralimbic regions greater in participants with a prominence of the inhibitory system over the activation system. Moreover, these neural differences are more evident in the *FTO* risk-allele AA genotype. The AA participants with higher anxiety and punishment sensitivity, reflected by a high BBr, showed greater connectivity in a dopaminergic cortico-striatal circuit including the insular cortex, associated with the perception of visceral states, and frontal areas such as the middle orbital gyrus and inferior frontal gyrus, which are involved in inhibitory control, decision making, and salience attribution. These findings suggest that in the at-risk AA allele carriers, higher anxiety scores are related to hyper-sensitivity to the hunger state, which in turn may lead to more frequent food consumption. Indeed, the AA participants with a high BBr, when compared to the AA participants with low BBr, also showed a greater connectivity in a network related to somatosensory integration regarding food, comprising the insular, olfactory, and opercularis cortices. The increased somatosensory processing of food properties might be responsible for the attitude toward fat-rich foods observed in these people. Moreover, the connectivity in the nigrostriatal circuit, involved in reward processing and avoidance learning, was increased in AA participants with lower BAS scores, supporting the hypothesis that impairment in avoidance-learning might be related to D2 receptor density in the striatum, which is lower in the carriers of the A allele. This hypothesis is also supported by the stronger relationship found in the AA group between the BBr and connectivity in the MTG (which is involved in addiction and in the processing of both drug and food cues) in all three networks.

Our work helps explain the complex interaction between genetics, neural patterns and behavioral measures in determining the risk for obesity. Understanding the neural correlates underpinning the association between one of the most studied obesity gene, such as the *FTO*, and the susceptibility to obesity, will help future research in developing individual-tailored strategies for obesity prevention.

## Author contributions

GO wrote the majority of the manuscript and performed most of the analysis. LW helped write the manuscript and perform analysis. EN, LS, AL, MO, VG, OT, and MB assisted with data collection as well as provided insight into the project and final manuscript. EN, LS, EL, CB, SB, and HS coordinated and planned the study in addition to developing original hypotheses. All authors have read and approve this version of the manuscript.

### Conflict of interest statement

The authors declare that the research was conducted in the absence of any commercial or financial relationships that could be construed as a potential conflict of interest.

## Abbreviations

*ACC*: anterior cingulate cortex
*BAS*: behavioral activation system
*BOLD*: blood oxygen level dependent
*BBr*: BIS/BAS ratio
*BIS*: behavioral inhibition system
*BMI*: body mass index
*CC*: cingulate cortex
*DARTEL*: Diffeomorphic Anatomical Registration Through Exponentiated Lie Algebra
*DMN*: default mode network
*DPARSFA*: Data Processing Assistant for Resting-state fMRI Advanced
*EEG*: electroencephalography
*EPI*: echo-planar imaging
*FSL*: *FMRIB* Software Library
*FWE*: family-wise error
*FWHM*: full width at half maximum
*GM*: gray matter
*GLM*: general linear model
*ICA*: independent component analysis
*IFG*: inferior frontal gyrus
*IPL*: inferior parietal lobule
*MAAS*: Mindfulness Attention Awareness Scale
*MCC*: middle cingulate cortex
*MELODIC*: Multivariate Exploratory Linear Optimized Decomposition into Independent Components
*MNI*: Neurological Institute
*MTG*: middle temporal gyrus
*PCC*: posterior cingulate cortex
*SMN*: sensorimotor network
*SN*: salience network
*SNPs*: single nucleotide polymorphisms
*SPL*: superior parietal lobule
*SPM*: Statistical Parametric Mapping
*SPSS*: Statistical Package for Social Science
*STG*: superior temporal gyrus
*TFCE*: threshold-free cluster enhancement
*TFE*: turbo-field-echo.
